# Sequences of Endophytic Fungal and Bacterial Communities from Araucaria araucana [(Molina) K. Koch, 1869] in the Coastal and Andes Mountain Ranges, Chile

**DOI:** 10.1128/MRA.00544-20

**Published:** 2020-07-02

**Authors:** Jaime Alarcón, Sebastián Márquez, Guus Teunisse, Carlos Mendoza, Claudio Meneses, Aida Baldini, Patricio Parra, Pablo Zamora, Freddy Boehmwald, Eduardo Castro-Nallar

**Affiliations:** a Universidad Andres Bello, Center for Bioinformatics and Integrative Biology (CBIB), Santiago, Chile; b Fundación Mar Adentro, Santiago, Chile; c Universidad Andres Bello, Centro de Biotecnología Vegetal (CBV), Santiago, Chile; d FONDAP Center for Genome Regulation, Santiago, Chile; e Corporación Nacional Forestal, Santiago, Chile; f Vincula S&C, Las Condes, Chile; g Done Properly Co., Santiago, Chile; University of California, Riverside

## Abstract

Here, we report the results from PCR and sequencing of bacterial 16S rRNA and fungal internal transcribed spacer 1 (ITS1) genes from needle, branch, trunk, and root samples of Araucaria araucana, plus soil and associated insects, collected along the entirety of its geographic distribution in Chile (January 2017 and 2018).

## ANNOUNCEMENT

Araucaria araucana (class Pinopsida, family Araucariaceae) is an endangered conifer with a fragmented and relict distribution in Chile and Argentina. *A. araucana* has been historically threatened by logging, wildfires, overgrazing, and extensive human harvesting of its seeds, which has pushed the species to the International Union for Conservation of Nature (IUCN) red list as an endangered species (1). *A. araucana* is regarded by the Chilean state as a national monument and by the native peoples of central and southern Chile as sacred.

Studies have shown that endophytic microbial communities play crucial roles in plant growth and fitness by supplying nutrients and protection against biotic and abiotic stress (2–5). However, few studies have characterized endophytic microbial communities in nonmodel plant species, such as *A. araucana*. Here, we report the results of 16S rRNA and internal transcribed spacer 1 (ITS1) amplicon sequencing of samples from needle, branch, trunk, root, and soil compartments and associated insects of *A. araucana* at 10 locations along most of its geographic range (Table 1) (1,325 samples total).

**TABLE 1:** Sequencing and taxonomic analysis results

Collection date^*a*^	Mountain range	Location^*b*^	16S rRNA				ITS				Coordinates	Mean altitude (masl^*c*^)	Sample sources^*d*^
			No. of sampled trees by location	No. of samples by location	No. of reads	No. of ASVs	No. of sampled trees by location	No. of samples by location	No. of reads	No. of ASVs			
January 2017	Andean	LM	6	28	620,100	3,914	6	28	636,836	1,768	37°53′47.7″S, 71°22′07.6″W	1,592	B, I, N, R, S, T
		LR	4	19	416,323	2,078	4	17	562,631	1,615	38°26′03.4″S, 71°28′41.3″W	1,586	B, I, N, R, S, T
		MC	13	61	1,501,519	6,355	13	61	1,627,559	3,480	38°25′13.1″S, 71°32′42.1″W	1,432	B, I, N, R, S, T
		MM	15	83	1,917,567	5,927	15	83	1,935,582	3,996	39°35′00.5″S, 71°27′44.5″W	1,209	B, I, N, R, S, T
		RC	15	98	2,299,030	5,874	15	99	2,458,879	3,900	37°56′06.1″S, 71°21′49.5″W	1,214	B, I, N, R, S, T
	Coastal	TG	14	90	1,685,186	4,710	14	86	2,325,777	4,382	37°41′44.7″S, 73°07′23.4″W	1,257	B, I, N, R, S, T
Average 2017			11.1	63.1	1,406,620.8	4,809.6	11.1	62.3	1,591,210.6	3,190.1			
January 2018	Andean	BP	8	29	761,399	716	8	29	370,560	679	39°27′25.1″S, 71°44′00.9″W	1,282	B, N, T
		CG	9	33	943,398	873	9	33	429,038	1,038	38°41′56.8″S, 71°49′14.2″W	1,323	B, N, T
		LM	9	34	932,893	1,014	8	25	346,203	590	37°53′48.6″S, 71°21′52.5″W	1,628	B, N, T
		LR	10	38	928,856	973	10	34	610,580	855	38°25′47.6″S, 71°27′47.1″W	1,618	B, N, T
		MC	8	30	837,521	601	8	27	422,978	705	38°25′20.9″S, 71°32′53.0″W	1,421	B, N, T
		RC	8	30	769,114	746	8	30	400,915	855	37°56′47.1″S, 71°19′56.6″W	1,078	B, N, T
		TH	8	30	795,112	1,070	8	29	491,550	1,027	38°11′58.8″S, 71°46′38.3″W	1,488	B, N, T
	Coastal	NB	10	36	953,189	1,438	10	35	627,473	1,151	37°48′20.3″S, 73°01′46.7″W	1,311	B, N, T
		TG	10	36	863,704	1,053	10	34	794,398	1,122	37°41′45.2″S, 73°07′20.4″W	1,274	B, N, T
Average 2018			8.9	32.8	865,020.6	942.6	8.7	30.6	499,299.4	891.3			
Total			147	675	16,224,911		146	650	14,040,959				

a We sampled 10 locations in 2 consecutive years (2017 and 2018).

b LM, La Mula; LR, Las Raíces; MC, Malalcahuello; MM, Mamuil Malal; RC, Ralco; TG, Trongol; BP, Bosque Pehuén; CG, Conguillío; TH, Tolhuaca; NB, Nahuelbuta.

c masl, meters above sea level.

d N, needle; B, branch; T, trunk; R, root; S, soil; I, insect.

Tissue samples were washed sequentially with 1.5 g/liter Captan (PubChem CID 8606), 70% ethanol, 1% sodium hypochlorite, and sterile water to remove epiphytic microbes. Then, plant material was ground manually before being flash frozen for 1 min (liquid nitrogen). After one cycle of tissue disruption in a TissueLyser II for 1 min, samples were frozen in liquid nitrogen again to repeat the disruption step. Fifty milligrams of disrupted plant material was used for DNA extraction using the DNeasy PowerPlant pro (Qiagen) extraction kit. Soil extractions were carried out using the PowerSoil DNA isolation kit (MoBio Laboratories). DNA was quantified by fluorimetry in a Qubit 3.0 instrument (Thermo Fisher Scientific) using the Qubit double-stranded DNA (dsDNA) high-sensitivity (HS) assay kit and stored at −80°C. For sequencing, we targeted the V4 region of the 16S rRNA gene and the ITS1 gene for taxonomic profiling in an Illumina MiSeq instrument (with an Illumina TruSeq kit; 2 × 250 and 2 × 150 bp for the 16S rRNA and ITS1 genes, respectively) (6–8). Samples were demultiplexed using the split_libraries_fastq.py module from QIIME 1.9 (16S, –barcode_type 12; ITS1, –rev_comp_barcode) (9), and amplicon sequence variants (ASVs) were inferred as in DADA2 v1.10.1 (10, 11) using the following parameters: maxEE = c(2); truncQ = 2; maxN, 0; and rm.phix, TRUE. Error rate learning, dereplication, and read merging were performed using default settings. Taxonomy was assigned using SILVA v132 and UNITE 01.12.2017 (tryRC = TRUE) (12, 13).

There were 16,224,911 reads from 16S rRNA and 14,040,959 reads for ITS1 from 675 and 650 samples, respectively (samples of <1,000 reads were not considered). *Proteobacteria* was the dominant phylum in all compartments, followed by *Actinobacteria* in needles, branches, and roots (8.1%, 4.9%, and 22.3%, respectively), and *Acidobacteria* (20.8%) and *Verrucomicrobia* (14.5%) in soil. *Firmicutes* and *Actinobacteria* comprised 10.4% and 10.3% relative abundance, respectively, in trunk samples. For the fungal samples, all compartments were dominated (>50% relative abundance) by Ascomycota, followed by Basidiomycota, with 28.7%, 20.5%, and 15.3% in needles, branches, and roots, respectively. Soil samples were characterized by high levels of Mortierellomycota (19.5%) and Mucoromycota (3.1%) compared with other compartments.

### Data availability.

Sequences from this data set are available through NCBI under the accession number PRJNA517193.
